# Supplementary material for: Nuclear Hsp104 safeguards the dormant translation machinery during quiescence
Source: Nat Commun. 2024 Jan 5;15:315. doi: 10.1038/s41467-023-44538-8 (PMC10770042; doi:10.1038/s41467-023-44538-8)
Supplement: Supplementary file 1 — Supplementary Information [file 41467_2023_44538_MOESM1_ESM.pdf]

Nuclear Hsp104 safeguards the dormant translation machinery during quiescence

Verena Kohler[1,2,3], Andreas Kohler[2,4,5], Lisa Larsson Berglund[6], Xinxin Hao[7], Sarah Gersing[8], Axel Imhof[9], Thomas Nyström[7], Johanna L. Höög[6], Martin Ott[4,10], Claes Andréasson[1,*], Sabrina Büttner[1,*]

[1] Department of Molecular Biosciences, The Wenner-Gren Institute, Stockholm University, 10691 Stockholm, Sweden
[2] Institute of Molecular Biosciences, University of Graz, 8010 Graz, Austria
[3] Department of Molecular Biology, Umeå University, 90187 Umeå, Sweden
[4] Department of Biochemistry and Biophysics, Stockholm University, 10691 Stockholm, Sweden
[5] Department of Medical Biochemistry and Biophysics, Umeå University, 90187 Umeå, Sweden
[6] Department of Chemistry and Molecular Biology, University of Gothenburg, 40530 Gothenburg, Sweden
[7] Department of Microbiology and Immunology, University of Gothenburg, 40530 Gothenburg, Sweden
[8] The Linderstrøm-Lang Centre for Protein Science, Department of Biology, University of Copenhagen, 1165 Copenhagen, Denmark
[9] Biomedical Center Munich, Faculty of Medicine, Ludwig Maximilian University of Munich, 82152 Planegg-Martinsried, Germany
[10] Department of Medical Biochemistry and Cell Biology, University of Gothenburg, 40530 Gothenburg, Sweden

Corresponding authors: claes.andreasson@su.se or sabrina.buettner@su.se

Supplementary Information

including:

Supplementary Figures 1-7

Supplementary Tables 1-3

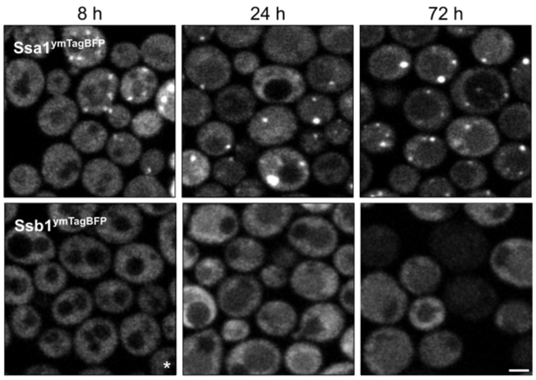

Supplementary Figure 1:

Hsp70 proteins are not re-directed to the nucleus when cells age.

Micrographs of BY4741 cells endogenously expressing ymTagBFP-tagged Ssa1 or Ssb1 at 8 h, 24 h and 72 h after inoculation. The signal intensity of Ssb1^{ymTagBFP} at 8 h was increased to ensure visibility (*). Scale bar: 2 µm.

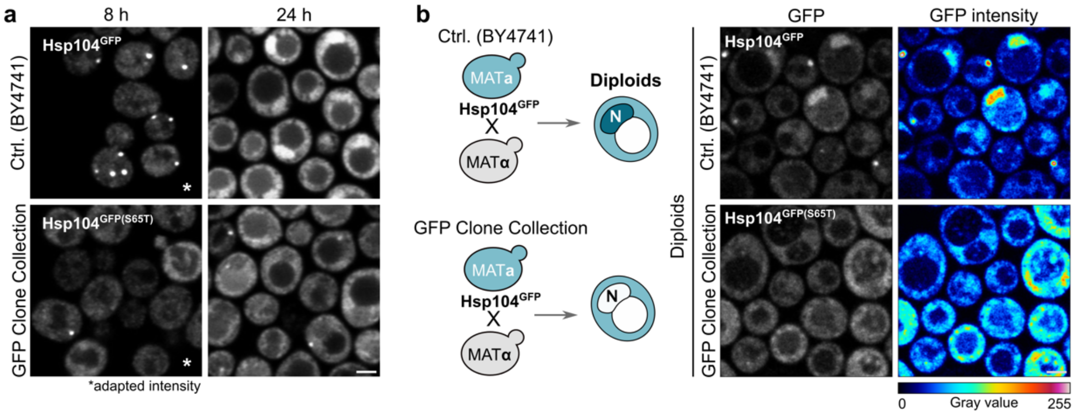

Supplementary Figure 2:

Nuclear targeting of Hsp104 depends on a motif in its flexible C-terminal tail.

(a) Micrographs of strains (BY4741 and BY4741 from the GFP Clone Collection) endogenously expressing Hsp104^{GFP} at 8 h and 24 h. The signal intensity of GFP-tagged Hsp104 at 8 h was increased to ensure visibility (*). Scale bar: 2 µm. **(b)** Micrographs of diploid yeast strains (simplified mating scheme depicted) endogenously expressing Hsp104^{GFP} at 24 h. Scale bar: 2 µm.

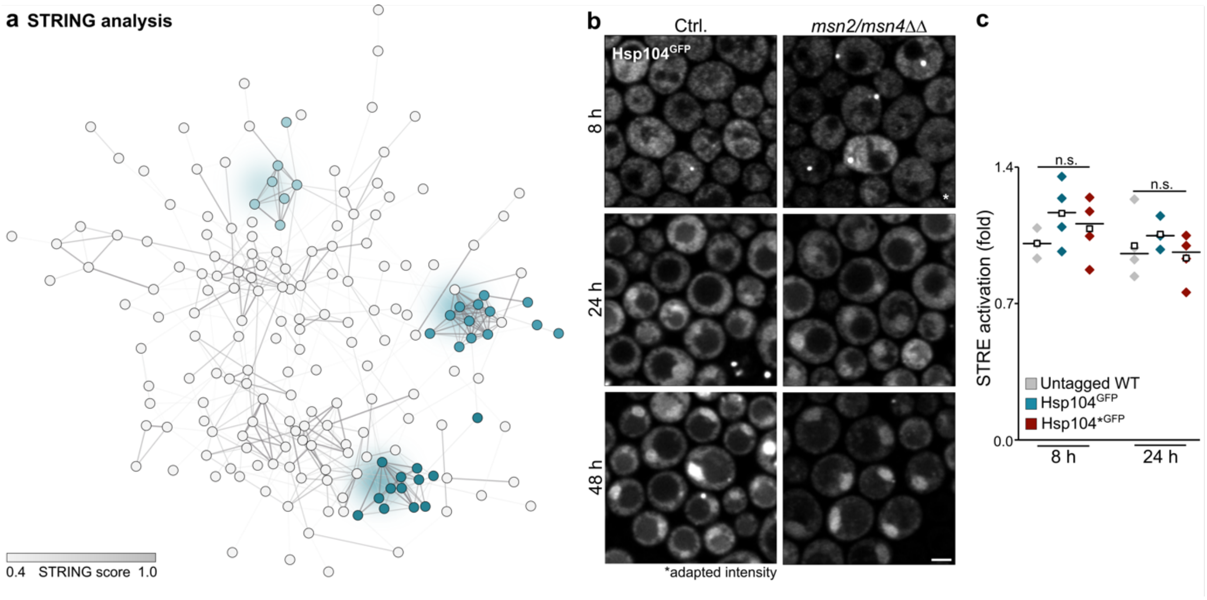

Supplementary Figure 3:

Mitochondrial respiration is critical for nuclear accumulation of Hsp104.

(a) STRING network of the hits scored as "weaker nuclear accumulation" in the microscopic screening of the genome-wide deletion library equipped with Hsp104^{GFP} (see Fig. 3a, b). Enrichment clusters depicted in Fig. 3b are highlighted. **(b)** Micrographs of wild type and *msn2/msn4*ΔΔ cells endogenously expressing Hsp104^{GFP} at 24 h. Scale bar: 2 µm. The signal intensity of GFP-tagged Hsp104 at 8 h was increased to ensure visibility (*). **(c)** Measurement of Msn2/4-induced stress response via activation of a STRE (stress response element)-driven promoter expressing Nanoluc in untagged wild type (WT) cells and cells with endogenously GFP-tagged Hsp104 variants at indicated time points. Dot plots with mean (square) and median (line). Each dot represents one biological replicate.

n.s.: not significant ($p \geq 0.05$). Source data are provided as a Source Data file. See Supplementary Table 3 for details on statistical analyses.

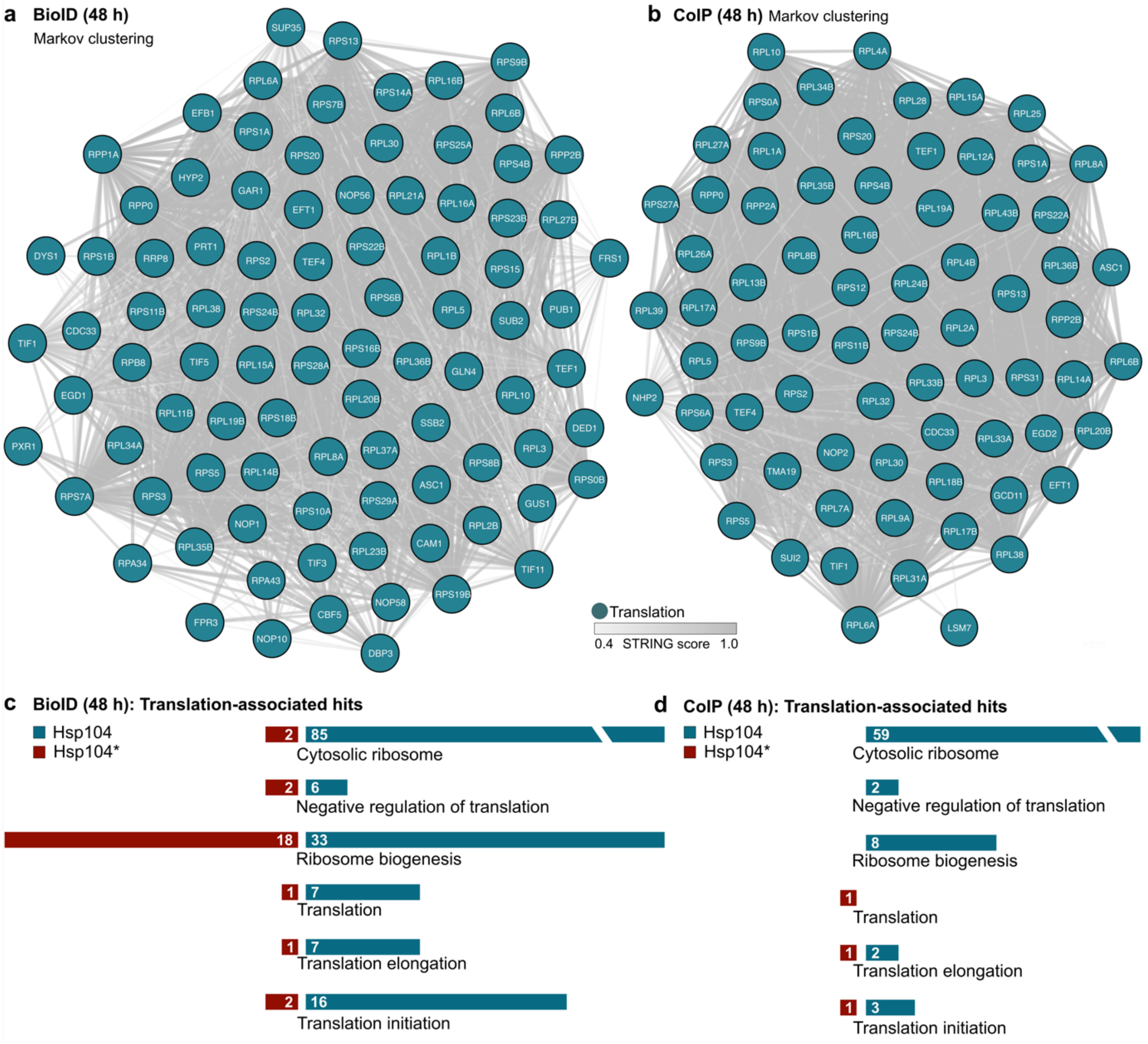

Supplementary Figure 4:

Nuclear Hsp104 interacts with translation-associated factors in aged cells.

(a) Visualization of genes associated with the term “Translation” as the most prominent enrichment cluster using Markov clustering from proximity labelling (BioID) experiments. **(b)** Visualization of genes associated with the term “Translation” as the most prominent enrichment cluster using Markov clustering from interaction (CoIP) experiments. See Fig. 4c, d for shared hits between CoIP and BioID after STRING analyses. **(c)** Classification of translation-associated hits from proximity labelling (BioID) experiments into different groups. Number of hits enriched in eluates from Hsp104 (turquoise) and Hsp104* (red) are shown. **(d)** Classification of translation-associated hits from interaction (CoIP) experiments into different groups. Number of hits enriched in eluates from Hsp104 (turquoise) and Hsp104* (red) are shown.

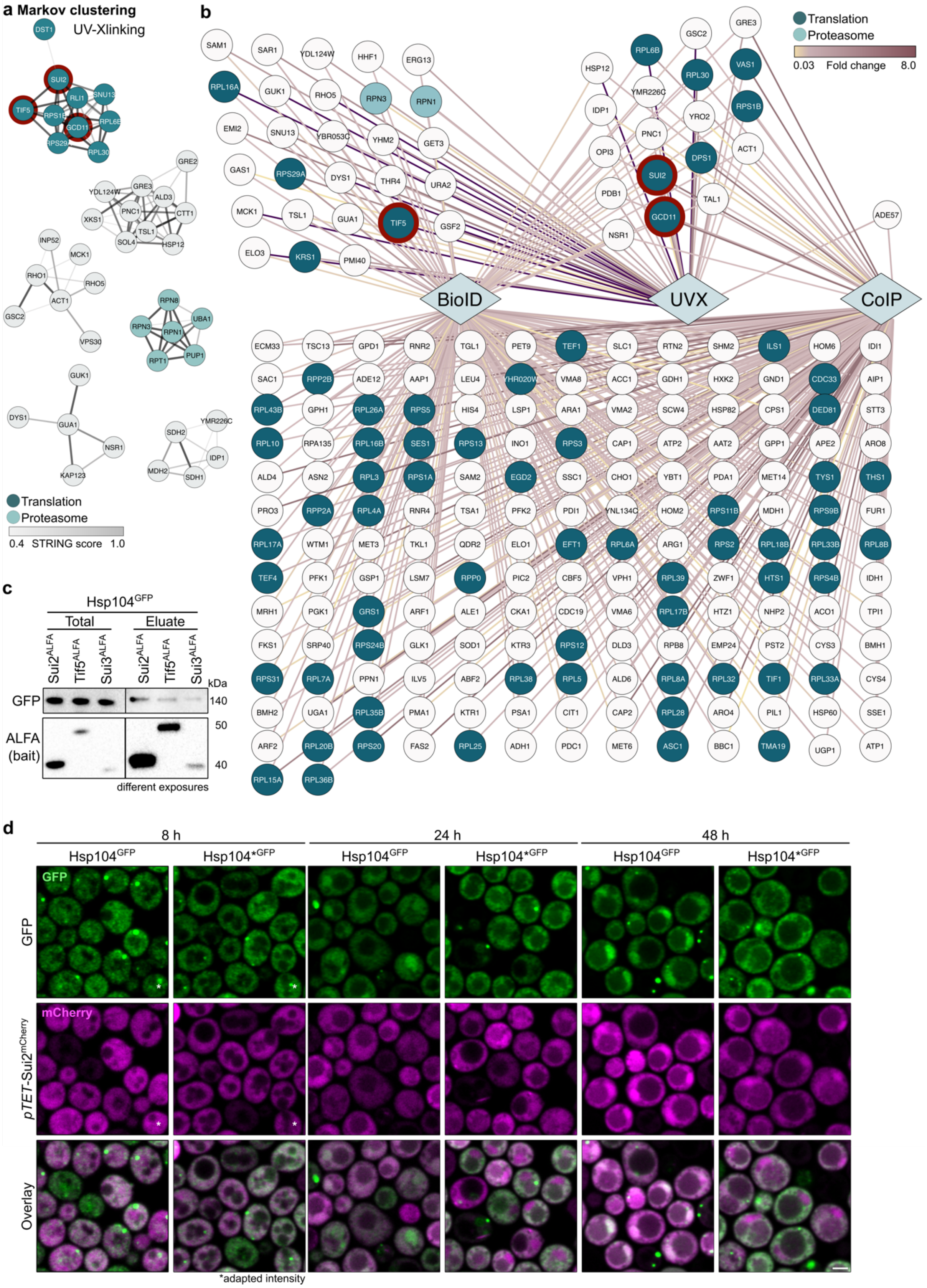
a Markov clustering
UV-Xlinking
DST1
SUI2
RLI1
SNU13
TIF5
RPS1B
GCD11
RPL6B
RPS29
RPL30
GRE2
YDL124W
GRE3
ALD3
XKS1
PNC1
CTT1
TSL1
SOL4
HSP12
INP52
MCK1
RHO1
RHO5
ACT1
GSC2
VPS30
RPN8
UBA1
RPN3
RPN1
RPT1
PUP1
GUK1
DYS1
GUA1
NSR1
KAP123
YMR226C
SDH2
IDP1
MDH2
SDH1
Translation
Proteasome
0.4 STRING score 1.0
b
Translation
Proteasome
0.03 Fold change 8.0
SAM1
SAR1
YDL124W
HHF1
ERG13
RPL16A
GUK1
RHO5
RPN3
RPN1
EMI2
SNU13
YBR053C
YHM2
GET3
GAS1
RPS29A
DYS1
THR4
URA2
MCK1
TSL1
GUA1
TIF5
GSF2
ELO3
KRS1
PMI40
GRE3
GSC2
RPL6B
HSP12
RPL30
VAS1
YMR226C
IDP1
RPS1B
YRO2
PNC1
OPI3
ACT1
DPS1
SUI2
PDB1
TAL1
GCD11
NSR1
ADE57
BioID
UVX
CoIP
ECM33
TSC13
GPD1
RNR2
TGL1
PET9
TEF1
SLC1
RTN2
SHM2
ILS1
HOM6
IDI1
SAC1
RPP2B
ADE12
AAP1
LEU4
YHR020W
VMA8
ACC1
GDH1
HXK2
GND1
CDC33
AIP1
RPL43B
GPH1
RPL26A
RPS5
HIS4
LSP1
ARA1
VMA2
SCW4
HSP82
CPS1
DED81
STT3
RPL10
RPA135
RPL16B
SES1
RPS13
INO1
RPS3
CAP1
ATP2
AAT2
GPP1
APE2
ARO8
ALD4
ASN2
RPL3
RPS1A
SAM2
EGD2
SSC1
CHO1
YBT1
PDA1
MET14
TYS1
THS1
PRO3
RPP2A
RPL4A
RNR4
TSA1
PFK2
PDI1
YNL134C
HOM2
RPS11B
MDH1
RPS9B
FUR1
RPL17A
WTM1
MET3
TKL1
QDR2
ELO1
EFT1
RPL6A
ARG1
RPS2
RPL18B
RPL33B
RPL8B
TEF4
PFK1
GSP1
LSM7
RPP0
PIC2
CBF5
VPH1
RPL39
ZWF1
HTS1
RPS4B
IDH1
MRH1
PGK1
GRS1
ARF1
ALE1
CKA1
CDC19
VMA6
RPL17B
HTZ1
NHP2
ACO1
TPI1
FKS1
SRP40
RPS24B
GLK1
SOD1
KTR3
RPS12
DLD3
RPB8
EMP24
PST2
CYS3
BMH1
RPS31
RPL7A
PPN1
ILV5
ABF2
RPL38
RPL5
ALD6
RPL8A
RPL32
TIF1
RPL33A
CYS4
BMH2
UGA1
RPL35B
PMA1
KTR1
PSA1
CIT1
CAP2
RPL28
ARO4
PIL1
HSP60
SSE1
ARF2
RPL20B
RPS20
FAS2
RPL25
ADH1
PDC1
MET6
ASC1
BBC1
TMA19
UGP1
ATP1
RPL15A
RPL36B
c
Hsp104GFP
Total
Eluate
Sui2ALFA
Tif5ALFA
Sui3ALFA
Sui2ALFA
Tif5ALFA
Sui3ALFA
kDa
GFP
140
ALFA
(bait)
50
40
different exposures
d
8 h
24 h
48 h
Hsp104GFP
Hsp104*GFP
Hsp104GFP
Hsp104*GFP
Hsp104GFP
Hsp104*GFP
GFP
GFP
mCherry
pTET-Sui2mCherry
Overlay
*adapted intensity

Supplementary Figure 5:

The translation initiation factor eIF2 interacts with nuclear Hsp104.

(a) Visualization of most prominent enrichment clusters of hits from UV-Xlinking experiments after STRING analysis and Markov clustering. See Fig. 5b for the complete STRING network. Hits associated with the terms "Translation" and "Proteasome" are highlighted, and the STRING score is depicted in shades of grey. **(b)** Network of hits enriched in Hsp104 eluates and identified in BioID (Fig. 4c, d), CoIP (Fig. 4c, d) and UV-Xlinking (Fig. 5b) experiments. Genes associated with the terms "Translation" and "Proteasome" are highlighted, and the fold change (Fc) is depicted (Fc values ≥ 1 in shades of red). **(c)** Immunoblot of pulldown assay using ALFA-tagged Sui2, Tif5 and Sui3 as baits. Blots were probed with antibodies directed against GFP and ALFA. Different exposure times are shown for Total and Eluate fractions. **(d)** Micrographs of cells expressing endogenously GFP-tagged Hsp104 variants and mCherry-tagged eIF2α/Sui2 under the control of a *TET*-promoter in its own locus (p*TET*-Sui2^{mCherry}), allowing its overexpression. The signal intensity of GFP-tagged Hsp104 at 8 h and mCherry-tagged Sui2 at 8 h were increased to ensure visibility (*). See Fig. 5d, e for quantification of 48 h. Scale bar: 2 µm. Source data are provided as a Source Data file. See Supplementary Table 3 for details on statistical analyses.

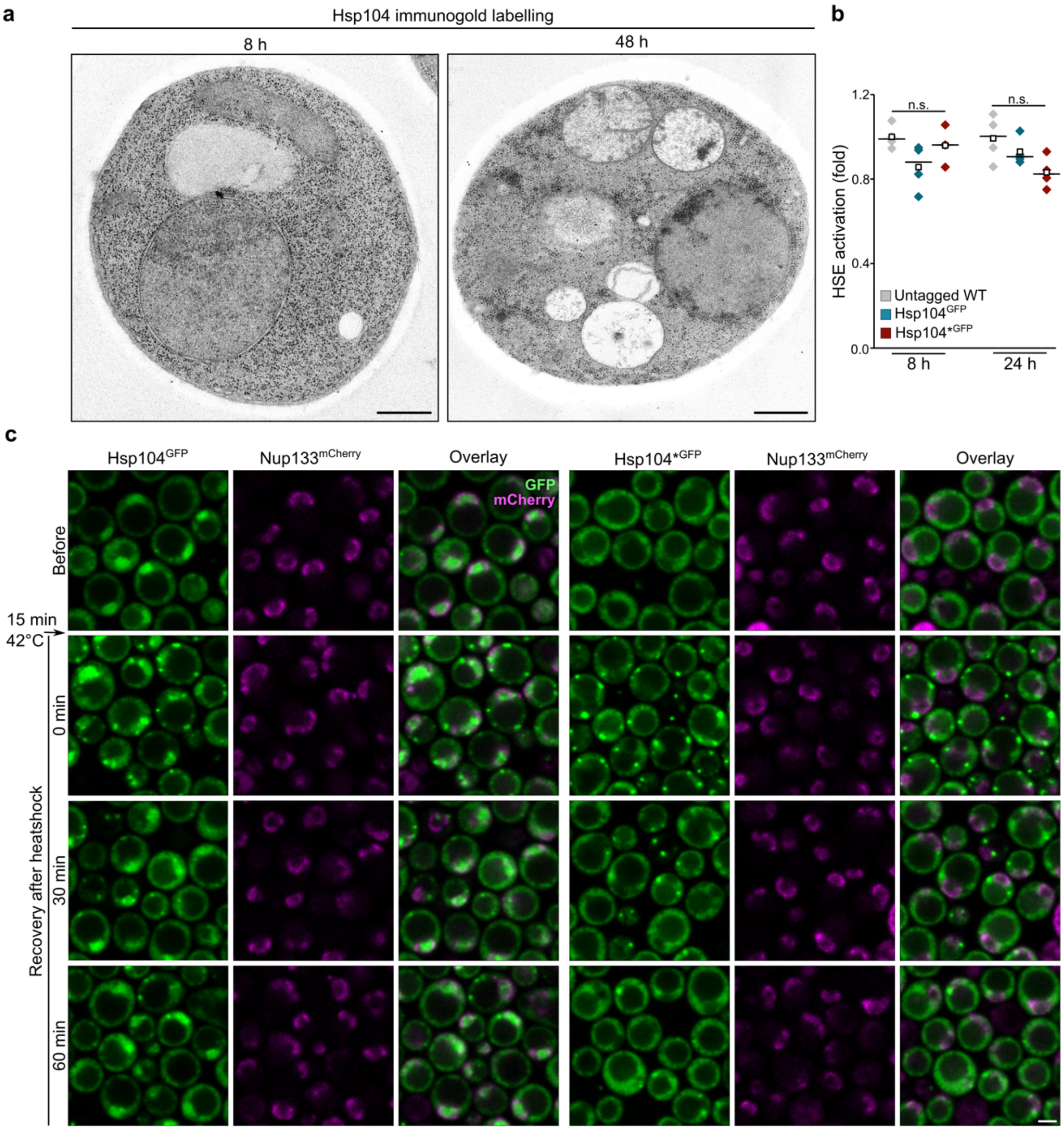

Supplementary Figure 6:

Hsp104 manages misfolded proteins in the nucleus of aged cells.

(a) Uncropped representative transmission electron micrographs of cells used for Hsp104 immunogold-labelling (Fig. 7b). Scale bar: 500 nm. **(b)** Measurement of heat stress response via activation of a HSE (heat shock element)-driven promoter expressing Nanoluc in untagged wild type (WT) cells and cells with endogenously GFP-tagged Hsp104 variants at indicated time points. Dot plots with mean (square) and median (line). Each dot represents one biological replicate. **(c)** Micrographs of cells harboring endogenously GFP-tagged Hsp104 wild type (Hsp104) or mutant variant (Hsp104*) and Nup133^{mCherry} to visualize the nucleus at 48 h before, during and after a heat shock at 42°C. Scale bar: 2 µm.
n.s.: not significant ($p \geq 0.05$). Source data are provided as a Source Data file. See Supplementary Table 3 for details on statistical analyses.

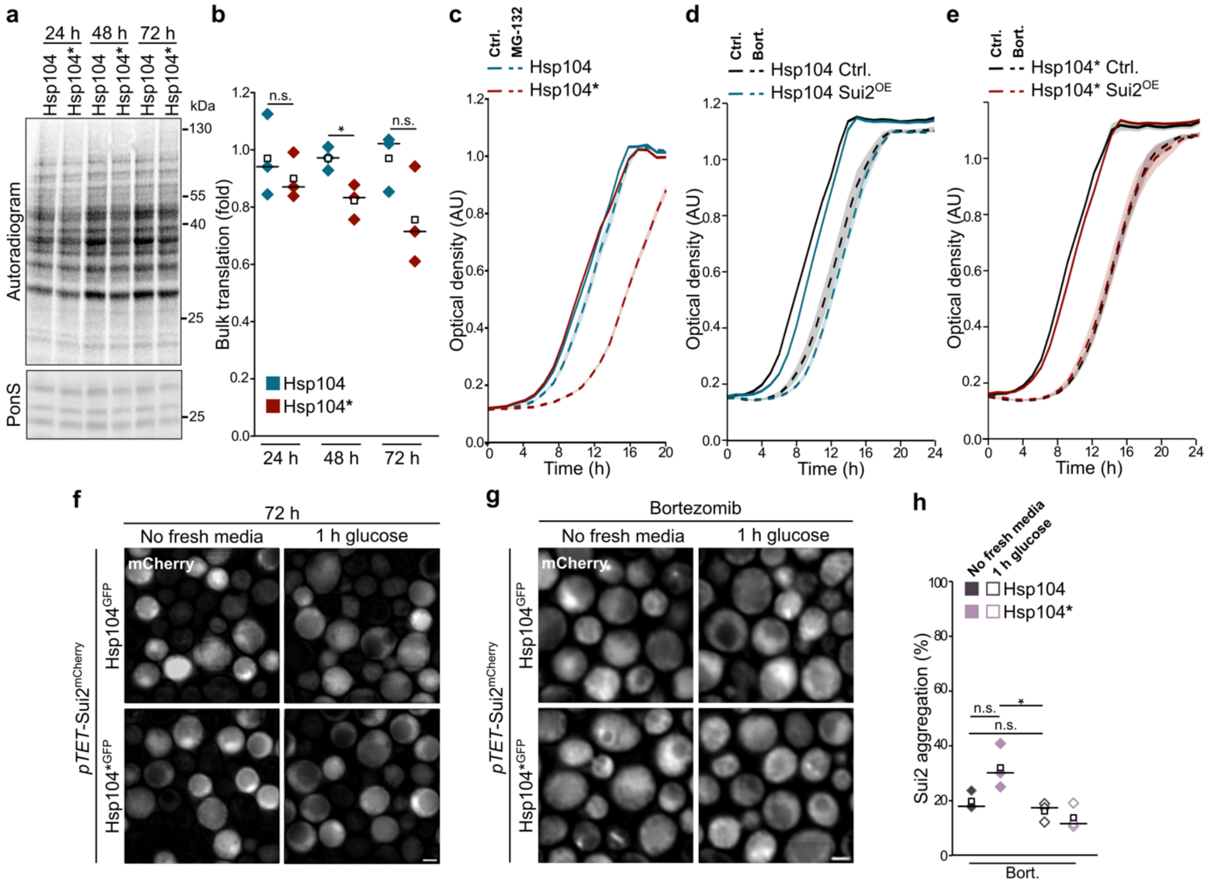

Supplementary Figure 7:

Nuclear Hsp104 ensures rapid restart of translation to support quiescence exit.

(a) [^{35}S]-methionine radiolabeling of nascent proteins from cells harboring untagged Hsp104 or Hsp104* for a time interval of 10 min. Radiolabeling was performed with cells at 24 h, 48 h or 72 h. Ponceau S (PonS) staining served as loading control. **(b)** Quantification of the autoradiogram from cells described in (a) is presented as fold values of wild type. Dot plots with mean (square) and median (line). Each dot represents one biological replicate. **(c)** Regrowth kinetics assessed as the optical density of cells (*pdr5*Δ) harboring untagged Hsp104 or Hsp104* cultured for 5 days. Cells were pre-treated with MG-132 at the first inoculation for proteasomal impairment as indicated. Line graph with mean ± s.e.m.. Measurements were taken from 9 biological replicates. **(d, e)** Regrowth kinetics assessed as the optical density of cells harboring endogenously GFP-tagged Hsp104 (d) or Hsp104* (e) cultured for 5 days. For overexpression of Sui2, cells expressing mCherry-tagged eIF2α/Sui2 under the control of a *TET*-promoter in its own locus (p*TET*-Sui2^{mCherry}) were used, allowing its overexpression. Cells were pre-treated with bortezomib at the first inoculation for proteasomal impairment as indicated. Line graph with mean ± s.e.m.. Measurements were taken from 12 biological replicates. **(f)** Micrographs of cells expressing mCherry-tagged eIF2α/Sui2 controlled by the *TET* promoter (p*TET*-Sui2^{mCherry}). At 72 h cells were shifted to fresh media 1 hour prior to analysis. Control conditions overlap with Fig. 5f, g.

Scale bar: 2 µm. **(g)** Micrographs of cells as described in (f) but with the addition of Bortezomib at initial inoculation. **(h)** Quantification of aggregation of Sui2 in cells as described in (g). Dot plots with mean (square) and median (line). Each dot represents one biological replicate (Bort. Ctrl - 72 h Hsp104: 738 cells, 72 h Hsp104*: 553 cells; Bort. Glucose – 72 h Hsp104: 785 cells, 72 h Hsp104*: 632 cells). Source data are provided as a Source Data file. See Supplementary Table 3 for details on statistical analyses.

Supplementary Table 1: Yeast strains used in this study.

Strain	Genotype	Source
**Introduced in Fig. 1**		
Ctrl. (BY4741)	MAT**a**, *his3*Δ1, *leu2*Δ0, *met15*Δ0, *ura3*Δ0	Euroscarf
Hsp104^{GFP}	BY4741, *HSP104*-yeGFP:kanMX	This study
Hsp82^{GFP}	BY4741, *HSP82*-yeGFP:kanMX	This study
Ssa4^{GFP}	BY4741, *SSA4*-yeGFP:kanMX	This study
Hsp104^{GFP} Nup133^{mCherry}	BY4741, *HSP104*-yeGFP:kanMX, *NUP133*-mCherry:hphNT1	This study
**Introduced in Fig. 2**		
S288c Hsp104^{GFP}	MAT**α,** *SUC2, gal2, mal2, mel, flo1, flo8-1, hap1, ho, bio1, bio6, HSP104*-yeGFP:kanMX	This study
W303 Hsp104^{GFP}	MAT**a,** *leu2-3,112, trp1-,1 can1-100, ura3-1, ade2-1, his3-11,15,* [phi+], *HSP104*-yeGFP:kanMX	This study
Hsp104^{GFP} (GFP Clone Coll.)	BY4741, *HSP104*-GFP(S85T):HIS3MX6	Thermofisher
Hsp104*^{GFP}	BY4741, *HSP104(M901V)*-yeGFP:kanMX	This study
Hsp104$^{\Delta CGFP}$	BY4741, *HSP104(900Δ)*-yeGFP:kanMX	This study
Hsp104*^{GFP} Nup133^{mCherry}	BY4741, *HSP104(M901V)*-yeGFP:kanMX, *NUP133*-mCherry:hphNT1	This study
p*TET*-Hsp104$_{aa901\text{-}908}^{GFP}$	BY4741, URA3:p*TET-HSP104(aa901-908*)-yeGFP:kanMX	This study
p*TET*-Hsp104*$_{aa901\text{-}908}^{GFP}$	BY4741, URA3:p*TET-HSP104(M901; aa901-908*)-yeGFP:kanMX	This study
**Introduced in Fig. 3**		
Hsp104^{GFP} deletion library	Gene deletion library mated with *MAT***α** *can1Δ::STE2pr-Sp_his5 lyp1Δ his3Δ1 leu2Δ0 ura3Δ0 met15Δ0 HSP104-GFP-LEU2*	This study
*cox12*Δ Hsp104^{GFP}	BY4741, *HSP104*-yeGFP:kanMX, *cox12*Δ::hphNT1	This study
*mip1*Δ Hsp104^{GFP}	BY4741, *HSP104*-yeGFP:kanMX, *mip1*Δ::hphNT1	This study
**Introduced in Fig. 4**		
Hsp104^{BirA*}	BY4741, *HSP104*-BirA*:HIS3MX6	This study
Hsp104*^{BirA*}	BY4741, *HSP104(M901V)*-BirA*:HIS3MX6	This study
**Introduced in Fig. 5**		
Hsp104(N897$_{TAG}$)^{ALFA}	BY4741, *trp1*Δ:hphNT2, *HSP104*(N897TAG)-ALFA:natNT2, ECYRS-BpA (Trp^{r})	This study
Hsp104(N897$_{TAG}$)*^{ALFA}	BY4741, *trp1*Δ:hphNT2, *HSP104*(N897TAG, *M901V*)-ALFA:natNT2, ECYRS-BpA	This study
Hsp104^{GFP} p*TET*-Sui2^{mCherry}	BY4741, *HSP104*-yeGFP:kanMX, URA3:p*TET-SUI2*-mCherry:hphNT1	This study
Hsp104*^{GFP} p*TET*-Sui2^{mCherry}	BY4741, *HSP104(M901V)*-yeGFP:kanMX, URA3:p*TET-SUI2*-mCherry:hphNT1	This study
**Introduced in Fig. 6**		
*pdr5*Δ Hsp104^{GFP}	BY4741, *HSP104*-yeGFP:kanMX, *pdr5*Δ:: hphNT1	This study
Hsp104^{GFP} p*GAL$_s$*-Sui2	BY4741, *HSP104*-yeGFP:kanMX, natNT2:p*GAL$_s$*-*SUI2*	This study
Hsp104^{GFP} p*GAL$_s$*-Tif5	BY4741, *HSP104*-yeGFP:kanMX, natNT2:p*GAL$_s$*-*TIF5*	This study
**Introduced in Fig. 7**		
Hsp104^{GFP} ^{GFP}FFL-NLS	BY4741, *HSP104*-yeGFP:kanMX, pCA924 (p*TDH3*-Luciferase-GFP-NLS (PKI) CEN/ARS URA3; Ap^{r})	This study
Hsp104*^{GFP} ^{GFP}FFL-NLS	BY4741, *HSP104(M901V)*-yeGFP:kanMX, pCA924	This study
*hsp104*Δ ^{GFP}FFL-NLS	BY4741, *hsp104*Δ:: hphNT1, pCA924	This study
*doa10*Δ*ubr1*Δ Hsp104^{GFP}	BY4741, *HSP104*-yeGFP:kanMX, *doa10*Δ:: hphNT1, *ubr1*Δ:: natNT2	This study
**Introduced in Fig. 8**		
*hsp104*Δ	BY4741, *hsp104*Δ:: hphNT1	This study

**Introduced in Supplementary Fig. 1**		
Ssa1^{ymTagBFP}	BY4741, *SSA1*-ymTagBFP:natNT2	This study
Ssb1^{ymTagBFP}	BY4741, *SSB1*-ymTagBFP:natNT2	This study
**Introduced in Supplementary Fig. 2**		
BY4742	MAT**α**, *his3*Δ1, *leu2*Δ0, *lys2*Δ0, *ura3*Δ0	Euroscarf
Diploid (Ctrl.)	BY4741xBY4742, *HSP104*-yeGFP:kanMX	This study
Diploid (GFP Clone Coll.)	BY4741xBY4742, *HSP104*-GFP(S65T):HIS3MX6	This study
**Introduced in Supplementary Fig. 3**		
*msn2/msn4*ΔΔ	BY4741, *MSN2*:: hphNT1, *MSN4*::natNT2, *HSP104*-yeGFP:kanMX	This study
WT p*STRE*-Nanoluc	BY4741, pAM17 (p$_{cyc}$*STRE*-Nanoluc CEN/ARS URA3; Ap^{r})	This study
Hsp104 p*STRE*-Nanoluc	BY4741, *HSP104*-yeGFP:kanMX, pAM17	This study
Hsp104* p*STRE*-Nanoluc	BY4741, *HSP104(M901V)*-yeGFP:kanMX, pAM17	This study
**Introduced in Supplementary Fig. 5**		
Sui2^{ALFA} Hsp104^{GFP}	BY4741, *SUI2*-ALFA:natNT2*, HSP104*-yeGFP:kanMX	This study
Tif5^{ALFA} Hsp104^{GFP}	BY4741, *TIF5*-ALFA:natNT2*, HSP104*-yeGFP:kanMX	This study
Sui3^{ALFA} Hsp104^{GFP}	BY4741, *SUI3*-ALFA:natNT2*, HSP104*-yeGFP:kanMX	This study
**Introduced in Supplementary Fig. 6**		
WT p*HSE*-Nanoluc	BY4741, pAM14 (p$_{cyc}$*HSE*-Nanoluc CEN/ARS URA3; Ap^{r})	This study
Hsp104 p*HSE*-Nanoluc	BY4741, *HSP104*-yeGFP:kanMX, pAM14	This study
Hsp104* p*HSE*-Nanoluc	BY4741, *HSP104(M901V)*-yeGFP:kanMX, pAM14	This study
**Introduced in Supplementary Fig. 7**		
Untagged Hsp104	BY4741, *HSP104*:kanMX, *pdr5*Δ:: hphNT1	This study
Untagged Hsp104*	BY4741, *HSP104(M901V)*:kanMX, *pdr5*Δ:: hphNT1	This study

Supplementary Table 2: Oligonucleotides used for gene deletion, chromosomal tagging and sequencing.

Modification	Oligonucleotide sequence (5'-3')	Template
Tagging of *HSP104*	TTATATTACTGATTCTTGTTCGAAAGTTTTTAAAAATCACACTAT<br>ATTAAATTAAATCGATGAATTCGAGCTCG<br>ACACGTTAGGTGATGACGATAATGAGGACAGTATGGAAATTGA<br>TGATGACCTAGACGTACGCTGCAGGTCGAC	pYM12, pYM44[1,2]
Combined construction and tagging of *HSP104**	TTATATTACTGATTCTTGTTCGAAAGTTTTTAAAAATCACACTAT<br>ATTAAATTAAATCGATGAATTCGAGCTCG<br>CACGTTAGGTGATGACGATAATGAGGACAGTGTGGAAATTGAT<br>GATGACCTAGATCGTACGCTGCAGGTCGAC	pYM12[1]
Combined construction and tagging of *HSP104*$^{\Delta C}$	TTATATTACTGATTCTTGTTCGAAAGTTTTTAAAAATCACACTATA<br>TTAAATTAAATCGATGAATTCGAGCTCG<br>TCACGAAGCTACTATAGGGGCTGACACGTTAGGTGATGACGATA<br>ATGAGGACAGTCGTACGCTGCAGGTCGAC	pYM12[1]
Construction of untagged *HSP104*	TTATATTACTGATTCTTGTTCGAAAGTTTTTAAAAATCACACTATA<br>TTAAATTAAATCGATGAATTCGAGCTCG<br>CACGTTAGGTGATGACGATAATGAGGACAGTATGGAAATTGATGA<br>TGACCTAGATTGACGTACGCTGCAGGTCGAC	pFA6a-natNT2[2]

Construction of untagged *HSP104**	TTATATTACTGATTCTTGTTCGAAAGTTTTTAAAAATCACACTATA TTAAATTAAATCGATGAATTCGAGCTCG<br>CACGTTAGGTGATGACGATAATGAGGACAGTGTGGAAATTGATGA TGACCTAGATTGACGTACGCTGCAGGTCGAC	pFA6a-natNT2[2]
Combined construction and tagging of *HSP104* for UV-Xlinking	TTATATTACTGATTCTTGTTCGAAAGTTTTTAAAAATCACACTATA TTAAATTAAATCGATGAATTCGAGCTCG<br>ACACGTTAGGTGATGACGATTAGGAGGACAGTATGGAAATTGAT GATGACCTAGATCGTACGCTGCAGGTCGAC	pYM17-ALFA
Combined construction and tagging of *HSP104** for UV-Xlinking	TTATATTACTGATTCTTGTTCGAAAGTTTTTAAAAATCACACTATAT TAAATTAAATCGATGAATTCGAGCTCG<br>ACACGTTAGGTGATGACGATTAGGAGGACAGTGTGGAAATTGAT GATGACCTAGATCGTACGCTGCAGGTCGAC	pYM17-ALFA
Deletion of *HSP104*	ATAACAAAGAAAAAAGAAATCAACTACACGTACCATAAAATATAC AGAATATATGCGTACGCTGCAGGTCGAC<br>TTATATTACTGATTCTTGTTCGAAAGTTTTTAAAAATCACACTATAT TAAATTAAATCGATGAATTCGAGCTCG	pFA6a-hphNT1[2]
Generation of $HSP104_{aa901\text{-}908}$ variants	AAAGAAAAAAGAAATCAACTACACGTACCATAAAATATACAGAAT ATATGCGTACGCTGCAGGTCGAC<br>AAGTTTTTAAAAATCACACTATATTAAATTAATCTAGGTCATCATC AATTTCCATCATCGATGAATTCTCTGTCG	pYM-N1-*TET*,
Tagging of *HSP82*	TACGTTATATTATGTTTTGTTTATAACCTATTCAAGGCCATGATGTT CTACCTAAATCGATGAATTCGAGCTCG<br>CCACCGCAGCTCCGGTTGAAGAGGTTCCAGCTGACACCGAAATGG AAGAGGTAGACGTACGCTGCAGGTCGAC	pYM12[1]
Tagging of *SSA4*	GGGAAAACTAAGAAATTCGATGCTGCTACTTCATCGCATCTTTGTA TTTATCTAAATCGATGAATTCGAGCTCG<br>CTGGAGCAGGCCCCACTGGAGCACCAGACAACGGCCCAACGGTTG AAGAGGTTGACGTACGCTGCAGGTCGAC	pYM12[1]
Tagging of *SSA1*	AGGTGGTGCTCCTCCAGCTCCAGAGGCTGAAGGTCCAACCGTTGAA GAAGTTGATCTTCGTACGCTGCAGGTCGA<br>CCCAGATCATTAAAAGACATTTTCGTTATTATCAATTGCCGCACCA ATTGGCTTACACTATAGGGAGACCGGCAG	pIM701
Tagging of *SSB1*	GAGAAAGGCAGAAGTTGGTTTGAAGAGAGTTGTCACCAAGGCCAT GTCTTCTCGTCTTCGTACGCTGCAGGTCGA<br>CATTAAAAATGAAAAATATATATATGTGTATAACCTTAACCAGAAT GACATCTTACACTATAGGGAGACCGGCAG	pIM701
Tagging of *NUP133*	TATCATTCCCCAGTAAAGTTTATTATATATATGTAAAATTGTATTAT AGATATTAATCGATGAATTCGAGCTCG<br>TGTAGCGAAAGAAAAAAACTATACCATCAACTATGAAACCAACAC TGTAGAATACCGTACGCTGCAGGTCGAC	pYM25-mCherry
Deletion of *COX12*	TGCTTTCACAAATAGGAACAAGCACATAAATACAGTATAATAGGC ATGCGTACGCTGCAGGTCGAC<br>AAAAAAGGCTAAGTGCACATTACTAAAAACGAAACTAAGATCCTT AATCGATGAATTCGAGCTCG	pFA6a-hphNT1[2]

Deletion of *MIP1*	GAAGAGGTCGAGATGGGGATTATATGTAGTTGTTGAGCAACGAGG GACAAGTATGCGTACGCTGCAGGTCGAC<br>TATAATGTGCTGTATATATAAATACAAATGCGAAAGCTAATGCAGA TTTTGCCTAATCGATGAATTCGAGCTCG	pFA6a-hphNT1[2]
Tagging of *SUI2*	CTAAAACAGTATGACACTTGAAAACACCTAGAAAAATTAGGCGCG GCAATGATTAATCGATGAATTCGAGCTCG<br>AGAATTAGATAATAGATCTGACTCTGAAGACGATGAGGATGAGTC AGACGACGAGCGTACGCTGCAGGTCGAC	pYM25-mCherry
Tagging of *TIF5*	TTACGCCATGTCATATAATAATTTACACATAAGTCTTTTGCCCCCTC CTAAGCTAATCGATGAATTCGAGCTCG<br>ATTCATTACGTGGTTAGAAACCGCTGAAAGTGACGATGATGAAGA AGACGACGAACGTACGCTGCAGGTCGAC	pYM17-ALFA
Tagging of *SUI3*	CGAAATCCGTATTTATTATATATATGCTAACAGGTAAAGCACCAAC ATCAATCGATGAATTCGAGCTCG<br>CTTCTATTAAAACCGGTTTCCAAGCTACCGTTGGTAAGAGAAGGAG AATGCGTACGCTGCAGGTCGAC	pYM17-ALFA
Promoter exchange of *SUI2*	TATTTTTGTGTCTTTTCTGCTGCCTCACGCACCTTCTATAATACACC AAATAATGCGTACGCTGCAGGTCGAC<br>CGATATCGTCAATTTCTGGGTATTTGTTTCATAAAATCTGCAATGA GAAGTGGACATCGATGAATTCTCTGTCG	pYM-N1-*TET*, pYM-N31[2]
Promoter exchange of *TIF5*	CCAAAAAAAAAAACTCCTATTATTTGCATAAACACACGTTCACTGG TACAAGATGCGTACGCTGCAGGTCGAC<br>GAGGCATTTTGTAACGGTAAAATGGATCATGATTATCTCTACAAAT ATTAATAGACATCGATGAATTCTCTGTCG	pYM-N31[2]
Deletion of *PDR5*	CCCTTTTAAGTTTTCGTATCCGCTCGTTCGAAAGACTTTAGACAAA AATGCGTACGCTGCAGGTCGAC<br>GTCCATCTTGGTAAGTTTCTTTTCTTAACCAAATTCAAAATTCTATT AATCGATGAATTCGAGCTCG	pFA6a-hphNT1[2]
Deletion of *MSN2*	TTTTTTCTTTCTTTTTTCAACTTTTATTGCTCATAGAAGAACTAGATC TAAAATG CGTACGCTGCAGGTCGAC<br>AACAGAATTATCTTATGAAGAAAGATCTATCGAATTAAAAAAATG GGGTCTATTAATCGATGAATTCGAGCTCG	pFA6a-hphNT1[2]
Deletion of *MSN4*	CGCCTTTATCAGTTCGGCTTTTTTTTCTTTTCTTCTTATTAAAAACAA TATAATGCGTACGCTGCAGGTCGAC<br>CTTGTCATACCGTAGCTTGTCTTGCTTTTATTTGCTTTTGACCTTATT TTTTTCAATCGATGAATTCGAGCTCG	pFA6a-natNT2[2]
Deletion of *DOA10*	TTTAGCCAAGAGTACCACTAATTGAATCAAAGAGACTAGAAGTGT GAAAGTCATGCGTACGCTGCAGGTCGAC<br>TATATATGTAAATATGCTAGCATTCATTTTAAATGTAAGGAAGAAA ACGCCTTTAATCGATGAATTCGAGCTCG	pFA6a-hphNT1[2]
Deletion of *UBR1*	ACTGAAGTCCCTAATCTTTACAGGTCACACAAATTACATAGAACAT TCCAATATGCGTACGCTGCAGGTCGAC<br>AAGTTTTTATATACAAATATGTCAACTATAAAACATAGTAGAGGGC TTGAATCTAATCGATGAATTCGAGCTCG	pFA6a-natNT2[2]

**Sequencing of Hsp104**	
Hsp104_-500	TTTCATGGTTTAAAAACCTTCTG
Hsp104_0	ATGAACGACCAAACGCAATTTAC
Hsp104_+500	GAATATTTATCAAAGTACGCCATTG
Hsp104_+1000	GAAAATTGAAGTCGCTGAACCAAG
Hsp104_+1500	AACGTAGATATGATACTGCTAC
Hsp104_+2000	CGATGAAGTAGAAAAGGCACATC
Hsp104_+2500	ACTAAGGATCTTAAAGAATGAAATC

Supplementary Table 3: Detailed description of statistical analyses performed in this study. Comparisons with p-values below 0.05 are marked in bold.

Fig.	Statistical test	Additional information	Results
**1e**	One-Way ANOVA with Tukey post hoc test	All assumptions met	F(3,8)= 130,9; p= 0.000000388;<br><br>**8 h vs. 24 h: p= 0.0000314;**<br>**8 h vs. 48 h: p= 0.000000632;**<br>**8 h vs. 72 h: p= 0.000000667;**<br>**24 h vs. 48 h: p= 0.000589;**<br>**24 h vs. 72 h: p= 0.000658;**<br>48 h vs. 72 h: p= 0.999;
**1f**	Kruskal Wallis test with a Wilcoxon signed rank test	Non-normally distributed data detected via Shapiro-Wilk test; Significantly different variances detected with Levene's test; Outlier detected	p= 1.205E-117;<br><br>**8 h vs. 24 h: p= 3.43969E-36;**<br>**8 h vs. 48 h: p= 8.07392E-63;**<br>**8 h vs. 72 h: p= 3.93957E-66;**<br>**24 h vs. 48 h: p= 3.1677E-32;**<br>**24 h vs. 72 h: p= 9.67145E-48;**<br>**48 h vs. 72h: p= 1.19161E-08;**
**1g**	Two-Way ANOVA mixed design with Bonferroni post hoc test	Outlier detected; Non-normally distributed data detected via Shapiro-Wilk test; Significantly different variances detected with Levene's test	Strain: F(2,297)= 24.9; p= 1.05E-10;<br>Time: F(1,297)= 88.1; p= 1.71E-18;<br>Strain: Time: F(2,297)= 26.8; p= 2.03E-11;<br><br>**48 h Cyto. vs. Mito.: p= 0.0000677;**<br>**48 h Cyto. vs. Nuc.: p = 0.000711;**<br>**48 h Mito. vs. Nuc.: p= 7E-14;**<br><br>8 h Cyto. vs. Mito.: p= 0.876;<br>8 h Cyto. vs. Nuc.: p= 0.962;<br>8 h Mito. vs. Nuc.: p= 0.913;<br><br>**Cyto. 8 h vs. 48 h: p= 9.52E-21;**<br>Mito.8 h vs. 48 h: p= 0.903;<br>**Nuc. 8 h vs. 48 h: p= 8.56E-11;**
**2b**	One-Way ANOVA with Tukey post hoc test	All assumptions met	F(3,8)= 234.5; p= 3.92E-08;<br><br>Ctrl. vs. S288c: p= 0.316;<br>**Ctrl. vs. W303: p= 0.0493;**<br>**Ctrl. vs. GFP Clone Coll.: p= 0.000000113;**<br>**S288c vs. W303: p= 0.00424;**<br>**S288c vs. GFP Clone Coll.: p= 0.000000269;**<br>**W303 vs. GFP Clone Coll.: p= 3.47E-08;**
**2e**	One-Way ANOVA with Tukey post hoc test	All assumptions met	F(2,6)= 335.99; p= 0.000000693;<br><br>**Hsp104 vs. Hsp104*: p= 0.00000126;**<br>**Hsp104 vs. Hsp104$^{\Delta C}$: p= 0.00000141;**<br>Hsp104* vs. Hsp104$^{\Delta C}$: p= 0.844;

**2h**	Two-Way ANOVA mixed design with Bonferroni post hoc test	Non-normally distributed data detected via Shapiro-Wilk test; Significantly different variances detected with Levene's test; Outlier detected	**Hsp104 Time F(1.61,164,8)= 164.8; p= 1.2E-22;**<br>**Hsp104* Time F(1.75, 205.3)= 73.3; p= 1E-35;**
**2j**	Wilcoxon-Mann-Whitney test	Outlier detected	**p=7.61E-43**
**3d**	One-Way ANOVA with Tukey post hoc test	All assumptions met	F(2,6)= 112.2; p= 0.0000177;<br><br>**Ctrl. vs. *cox12*Δ: p= 0.0000296;**<br>**Ctrl. vs. *mip1*Δ: p= 0.0000343;**<br>*cox12*Δ vs. *mip1*Δ: p= 0.944;
**3g**	Two-tailed student T-test	All assumptions met	**Glucose vs. glycerol: p=0.010979869;**
**5e**	Wilcoxon-Mann-Whitney test	Outlier detected	**p=7.61E-43**
**5g**	Two-Way ANOVA mixed design with Bonferroni post hoc test	Normally distributed data detected via Shapiro-Wilk test; No significantly different variances detected with Levene's test; Outlier detected	Strain (24h) F(1,10)=3.494; p=0.273;<br>Strain (48h) F(1,10)=2.726; p=0.39;<br>Strain (72h) F(1,10)=35.5; p=0.000438;<br><br>Time (Hsp104) F(2,10)=50.613; p=0.00001174;<br><br>24h Hsp104 vs. Hsp104*: p=0.0911;<br>48h Hsp104 vs. Hsp104*: p=0.13;<br>**72h Hsp104 vs. Hsp104*: p=0.000146**
**5h**	Two-Way ANOVA mixed design with Bonferroni post hoc test	Non-normally distributed data detected via Shapiro-Wilk test; Significantly different variances detected with Levene's test; Outlier detected	Strain (d1) F(1,10)=5.102; p=0.141;<br>Strain (d2) F(1,10)=0.016; p=1;<br>Strain (d3) F(1,10)=1.527;p=0.132<br><br>**D1 Hsp104 vs. Hsp104*: p=0.0475;**<br>D2 Hsp104 vs. Hsp104*: p=0.901;<br>D3 Hsp104 vs. Hsp104*: p=0.245
**6b**	Ctrl.: Two-tailed student T-test	All assumptions met	Galactose vs. glucose: p=0.15982
**6b**	Sui2: Two-tailed student T-test	All assumptions met	**Galactose vs. glucose: p= 0.000176711**

**6b**	Tif5: Wilcoxon-Mann-Whitney test	Outlier detected	**Galactose vs. glucose: p=0.0286**
**6d**	Kruskal Wallis test with a Wilcoxon signed rank test	Non-normally distributed data detected via Shapiro-Wilk test; Not significantly different variances detected with Levene's test; Outlier detected	p=8.85E-10;<br><br>**WT Ctrl. vs. WT 1 h Gluc.: p=0.00000187;**<br>WT Ctrl. vs. Sui2 Ctrl: p=0.996;<br>WT Ctrl. vs. Sui2 1 h Gluc.: p=1;<br>WT Ctrl. vs. Tif5 Ctrl.: p=0.075;<br>WT Ctrl vs. Tif5 1 h Gluc.: p=0.999;<br>**WT 1 h Gluc. vs. Sui2 Ctrl.: p=0.0000158;**<br>**WT 1 h Gluc. vs. Sui2 1 h Gluc.: p=0.00000118;**<br>WT 1 h Gluc. vs. Tif5 Ctrl.: p=0.067;<br>**WT 1 h Gluc. vs. Tif5 1 h Gluc.: p=0.000000036;**<br>Sui2 Ctrl. vs. Sui2 1 h Gluc.: p=0.999;<br>Sui2 Ctrl. vs. Tif5 Ctrl.: p=0.225;<br>Sui2 Ctrl. vs. Tif5 1 h Gluc.: p=0.949;<br>Sui2 1 h Gluc. vs. Tif5 Ctrl.: p=0.082;<br>Sui2 1 h Gluc. vs. Tif5 1 h Gluc.: p=0.995;<br>**Tif5 Ctrl. vs. Tif5 1 h Gluc.: p=0.029**
**7b**	Two-Way ANOVA mixed design with Bonferroni post hoc test	Non-normally distributed data detected via Shapiro-Wilk test; Significantly different variances detected with Levene's test; Outlier detected	Strain: F(1,198)= 25.6; p= 0.000000968;<br>Time: F(1,198)= 50.2; p= 2.34E-11;<br>Strain: Time: F(1,198)= 33.3; p= 0.00000003;<br><br>**48 h Nucleoplasm vs. Nuclear EDC: p= 0.000000332;**<br>**8 h Nucleoplasm vs. Nuclear EDC: p= 0.000001212;**<br>**Nucleoplasm 8 h vs. 48 h: p=5.48E-09;**<br>**Nuclear EDC 8 h vs. 48 h: p=0.00091**
**7j**	Wilcoxon-Mann-Whitney test	Outlier detected	p= 0.0000452
**8c**	Kruskal Wallis test with a Wilcoxon signed rank test	Non-normally distributed data detected via Shapiro-Wilk test; Significantly different variances detected with Levene's test; Outlier detected	p= 3.59126E-08;<br><br>Ctrl. Hsp104 vs. Ctrl. Hsp104*: p=0.590827261;<br>**Ctrl. Hsp104 vs. MG-132 Hsp104: p= 0.000663766;**<br>**Ctrl. Hsp104 vs. MG-132 Hsp104*: p= 0.000663766;**<br>Ctrl. Hsp104 vs. Ctrl. *hsp104*Δ: p=0.259804305;<br>**Ctrl. Hsp104 vs. MG-132 *hsp104*Δ: p= 0.000663766;**<br>**MG-132 Hsp104 vs. Ctrl. Hsp104*: p=0.000663766;**<br>**MG-132 Hsp104 vs. MG-132 Hsp104*: p=0.019070284;**<br>**MG-132 Hsp104 vs. Ctrl. *hsp104*Δ: p=0.000663766;**<br>**MG-132 Hsp104 vs. MG-132 *hsp104*Δ: p= 0.005436823;**<br>**Ctrl. Hsp104* vs. MG-132 Hsp104*: p= 0.000663766;**<br>Ctrl. Hsp104* vs. Ctrl. *hsp104*Δ: p=0.569558354;<br>**Ctrl. Hsp104* vs. MG-132. *hsp104*Δ: p= 0.000663766;**<br>**MG-132 Hsp104* vs. Ctrl. *hsp104*Δ: p= 0.000663766;**

			MG-132 Hsp104* vs. MG-132. *hsp104*Δ: p= 0.208877579;<br>**Ctrl. *hsp104*Δ vs. MG-132 *hsp104*Δ: p=0.000663766**
**8e<br>48 h**	Kruskal Wallis test with a Wilcoxon signed rank test	Outlier detected	p= 0.0039685;<br><br>Ctrl. Hsp104 vs. Ctrl Hsp104*: p=0.371428571;<br>**Ctrl. Hsp104 vs. Gluc. Hsp104: p=0.006493506;**<br>Ctrl. Hsp104 vs. Gluc. Hsp104*: p=0.371428571;<br>Ctrl. Hsp104* vs. Gluc. Hsp104: p=0.017316017;<br>Ctrl. Hsp104* vs. Gluc. Hsp104*: p=0.48484848;<br>**Gluc. Hsp104 vs. Gluc Hsp104*: p=0.00649351**
**8e<br>72 h**	Kruskal Wallis test with a Wilcoxon signed rank test	Outlier detected	p=0.001617;<br><br>**Ctrl. Hsp104 vs. Ctrl. Hsp104*: p=0.004329004;**<br>**Ctrl. Hsp104 vs. Gluc. Hsp104: p=0.004329004;**<br>**Ctrl. Hsp104 vs. Gluc. Hsp104*: p=0.004329004;**<br>Ctrl. Hsp104* vs. Gluc. Hsp104: p=0.13961039;<br>Ctrl. Hsp104* vs. Gluc. Hsp104*: p=0.288311688;<br>Gluc. Hsp104 vs. Gluc Hsp104*: p=0.30952381
**8g**	One-Way ANOVA with Tukey post hoc test	All assumptions met	F(3,20)=8.886; p=0.000605<br><br>**Ctrl. Hsp104 vs. Ctrl. Hsp104*: p=0.000916;**<br>Ctrl. Hsp104 vs. Gluc. Hsp104: p=0.987;<br>Ctrl. Hsp104 vs. Gluc. Hsp104*: p=0.639;<br>**Ctrl. Hsp104* vs. Gluc. Hsp104: p=0.00195;**<br>**Ctrl. Hsp104* vs. Gluc. Hsp104*: p=0.0136;**<br>Gluc. Hsp104 vs. Gluc. Hsp104*: p=0.824
**Suppl. 3c**	8 h: One-Way ANOVA with Tukey post hoc test	All assumptions met	F(2,9)=1.211; p=0.342;<br><br>Untagged WT vs. Hsp104: p=0.312;<br>Untagged WT vs Hsp104*: p=0.736;<br>Hsp104 vs. Hsp104*: p=0.714
**Suppl. 3c**	24 h: Kruskal Wallis test with a Wilcoxon signed rank test	Outlier detected	p=0.389737;<br><br>Untagged WT vs Hsp104: p=0.72857;<br>Untagged WT vs. Hsp104*: p=1;<br>Hsp104 vs. Hsp104*: p=0.6
**Suppl. 6b**	8 h: Kruskal Wallis test with a Wilcoxon signed rank test	Outlier detected	p=0.069039;<br><br>Untagged WT vs. Hsp104: p=0.17142;<br>Untagged WT vs. Hsp104*: p=0.4857;<br>Hsp104 vs. Hsp104*: p=0.17142
**Suppl. 6b**	24 h: Kruskal Wallis test with a Wilcoxon signed rank test	Outlier detected	p=0.09212;<br><br>Untagged WT vs. Hsp104: p=0.48571;<br>Untagged WT vs Hsp104*: p=0.17142;<br>Hsp104 vs. Hsp104*: p=0.3
**Suppl. 7b**	24 h: Two-tailed student T-test	All assumptions met	p=0.499477
**Suppl. 7b**	48 h: Two-tailed student T-test	All assumptions met	p=0.02545
**Suppl. 7b**	72 h: Two-tailed student T-test	All assumptions met	p= 0.133245826
**Suppl. 7h**	One-Way ANOVA with Tukey post hoc test	All assumptions met	F(3,8)= 7.143; p= 0.012;<br><br>Ctrl. Hsp104 vs. Ctrl. Hsp104*: p=0.0816;<br>Ctrl. Hsp104 vs. Gluc. Hsp104: p=0.834;

	Ctrl. Hsp104 vs. Gluc. Hsp104*: p=0.534;
	**Ctrl. Hsp104* vs. Gluc. Hsp104: p=0.0252;**
	**Ctrl. Hsp104* vs. Gluc. Hsp104*: p=0.0119;**
	Gluc. Hsp104 vs. Gluc. Hsp104*: p=0.942

References

1. Knop, M. et al. Epitope tagging of yeast genes using a PCR-based strategy: more tags and improved practical routines. Yeast 15, 963–972 (1999).

2. Janke, C. et al. A versatile toolbox for PCR-based tagging of yeast genes: new fluorescent proteins, more markers and promoter substitution cassettes. Yeast 21, 947–962 (2004).
