# Supplementary material for: Nuclear Hsp104 safeguards the dormant translation machinery during quiescence
Source: Nat Commun. 2024 Jan 5;15:315. doi: 10.1038/s41467-023-44538-8 (PMC10770042; doi:10.1038/s41467-023-44538-8)
Supplement: Supplementary file 3 — Reporting Summary [file 41467_2023_44538_MOESM3_ESM.pdf]

nature portfolio

Corresponding author(s): Sabrina Büttner

Last updated by author(s): Dec 7, 2023

Reporting Summary

Nature Portfolio wishes to improve the reproducibility of the work that we publish. This form provides structure for consistency and transparency in reporting. For further information on Nature Portfolio policies, see our Editorial Policies and the Editorial Policy Checklist.

Statistics

For all statistical analyses, confirm that the following items are present in the figure legend, table legend, main text, or Methods section.

n/a	Confirmed	
☐	☒	The exact sample size (*n*) for each experimental group/condition, given as a discrete number and unit of measurement
☐	☒	A statement on whether measurements were taken from distinct samples or whether the same sample was measured repeatedly
☐	☒	The statistical test(s) used AND whether they are one- or two-sided<br>*Only common tests should be described solely by name; describe more complex techniques in the Methods section.*
☒	☐	A description of all covariates tested
☐	☒	A description of any assumptions or corrections, such as tests of normality and adjustment for multiple comparisons
☐	☒	A full description of the statistical parameters including central tendency (e.g. means) or other basic estimates (e.g. regression coefficient) AND variation (e.g. standard deviation) or associated estimates of uncertainty (e.g. confidence intervals)
☐	☒	For null hypothesis testing, the test statistic (e.g. *F*, *t*, *r*) with confidence intervals, effect sizes, degrees of freedom and *P* value noted<br>*Give P values as exact values whenever suitable.*
☒	☐	For Bayesian analysis, information on the choice of priors and Markov chain Monte Carlo settings
☒	☐	For hierarchical and complex designs, identification of the appropriate level for tests and full reporting of outcomes
☒	☐	Estimates of effect sizes (e.g. Cohen's *d*, Pearson's *r*), indicating how they were calculated

Our web collection on statistics for biologists contains articles on many of the points above.

Software and code

Policy information about availability of computer code

Data collection	For data collection, the following softwares and codes were used: ZEN blue software (ZEISS), IMOD, ChemiDocXRS+Imaging System (Bio-Rad), Bioscreen CTM Software, Orion II Microplate Luminometer Software
Data analysis	Data were analyzed and graphs were generated with Fiji, IMOD, Image Lab 5.2.1 Software (Bio-Rad), Cytoscape (v. 3.9.1), Github (ddamsproteomics vs2.7, LIMMA-pipeline-proteomics), R Studio (v.1.4.17.17; with the following packages: ggplot2, ggpubr, car, rstatix, tidyverse) and InkScape (v.1.2.2).

For manuscripts utilizing custom algorithms or software that are central to the research but not yet described in published literature, software must be made available to editors and reviewers. We strongly encourage code deposition in a community repository (e.g. GitHub). See the Nature Portfolio guidelines for submitting code & software for further information.

Data

Policy information about availability of data

All manuscripts must include a data availability statement. This statement should provide the following information, where applicable:

- Accession codes, unique identifiers, or web links for publicly available datasets
- A description of any restrictions on data availability
- For clinical datasets or third party data, please ensure that the statement adheres to our policy

Mass spectrometry data generated in this study have been deposited in the ProteomeXchange Consortium database via the PRIDE partner repository under the

accession code PXD042987 [https://www.ebi.ac.uk/pride/archive?keyword=PXD042987] for the BioID-MS data and under PXD043093 [https://www.ebi.ac.uk/pride/archive?keyword=PXD043093] for the CoIP-MS data as well as UV-Crosslink-MS data. Source data are provided with this paper.

Research involving human participants, their data, or biological material

Policy information about studies with human participants or human data. See also policy information about sex, gender (identity/presentation), and sexual orientation and race, ethnicity and racism.

Reporting on sex and gender	Not applicable, as yeast has been used as sole model organism.
Reporting on race, ethnicity, or other socially relevant groupings	Not applicable, as yeast has been used as sole model organism.
Population characteristics	Not applicable, as yeast has been used as sole model organism.
Recruitment	Not applicable, as yeast has been used as sole model organism.
Ethics oversight	Not applicable, as yeast has been used as sole model organism.

Note that full information on the approval of the study protocol must also be provided in the manuscript.

Field-specific reporting

Please select the one below that is the best fit for your research. If you are not sure, read the appropriate sections before making your selection.

☒ Life sciences ☐ Behavioural & social sciences ☐ Ecological, evolutionary & environmental sciences

For a reference copy of the document with all sections, see nature.com/documents/nr-reporting-summary-flat.pdf

Life sciences study design

All studies must disclose on these points even when the disclosure is negative.

Sample size

For all experiments at least three biological replicates (different yeast clones, inoculated into separate culture flasks) were used. Additionally, for quantifications of confocal and transmission electron micrographs, the total number of cells per condition/time point is given in the respective figure legend. The use of a minimum of three biological replicates has been empirically determined by pilot experiments.

In our studies, we used a minimum of three biological replicates for the following reasons:
Statistical Robustness: We aimed to calculate measures of central tendency and dispersion, such as the mean and standard deviation, to provide a more accurate representation of our data.
Biological Variation: We recognized the inherent variability in biological systems. By using multiple biological replicates (refering to different yeast clones), we ensured that the observed effects were not due to random chance but were a true representation of the underlying biology.
Practical Considerations: Our choice of biological replicates was also influenced by practical considerations such as cost, time, and resources. We considered three as a minimum number where we could start to make meaningful statistical inferences without overstretching resources.
While we acknowledge that increasing the number of replicates could improve the reliability and power of our study, we balanced the need for statistical power with the practical constraints of our study.

Data exclusions

No data was excluded from the analysis.

Replication

Data shown in the manuscript were obtained from representative experiments, each performed with at least three biological replicates. Biochemical experiments including confocal microscopy were replicated at least three times and all attempts at replication were successful. Details provided in the Methods section.

Randomization

Not relevant to this study. The study does not involve participant groups. Each experiment is based on randomly selected clones from the same genetic background under identical culturing conditions, performed as described in the Methods section in detail.

Blinding

In our yeast studies, we did not use blinding due to the following reasons:
Strong Phenotype: We studied a strong phenotype that was easily distinguishable and unlikely to be influenced by bias or subjective interpretation.
Unbiased High Throughput Studies: Our experiments were high throughput, meaning they were largely automated and/or unbiased and involved minimal human intervention, reducing the potential for bias.
Complexity: Blinding added complexity to the experimental design. In our case, the simplicity and clarity of our experimental design was a priority.

Reporting for specific materials, systems and methods

We require information from authors about some types of materials, experimental systems and methods used in many studies. Here, indicate whether each material, system or method listed is relevant to your study. If you are not sure if a list item applies to your research, read the appropriate section before selecting a response.

Materials & experimental systems

n/a	Involved in the study	
☐	☒	Antibodies
☒	☐	Eukaryotic cell lines
☒	☐	Palaeontology and archaeology
☐	☒	Animals and other organisms
☒	☐	Clinical data
☒	☐	Dual use research of concern
☒	☐	Plants

Methods

n/a	Involved in the study	
☒	☐	ChIP-seq
☒	☐	Flow cytometry
☒	☐	MRI-based neuroimaging

Antibodies

Antibodies used	For immunoblotting: monoclonal anti-GFP (dilution 1:2 500, mouse, Sigma-Aldrich, Cat#1814460001); monoclonal anti-tubulin (dilution 1:1 0000, rabbit, Abcam, Cat#ab184970); polyclonal anti-Mouse IgG (whole molecule)–Peroxidase (dilution 1:10 000, rabbit, Sigma-Aldrich, Cat#A9044); polyclonal anti-Rabbit IgG (whole molecule)-Peroxidase (dilution 1:10 000, goat, Sigma-Aldrich, Cat#A0545);<br>For TEM immunogold-labelling: anti-Hsp104 (dilution 1:100, Abcam, rabbit, ab69549); 10 nm gold-labeled Goat-anti-Rabbit IgG (H&L) (dilution 1:20, Electron Microscopy, Cat#25108)
Validation	anti-GFP was validated by the manufacturer (Sigma-Aldrich) and has been referenced in several publications (see manufacturer's homepage); anti-Tubulin was validated by the manufacturer (Abcam) and has been referenced in 15 publications (see manufacturer's homepage); both secondary HRP-conjugated antibodies were validated by the manufacturer (Sigma-Aldrich) and have been referenced in several publications (see manufacturer's homepage);<br>Both antibodies used for Immunogold-EM experiments were extensively validated by J. Höög's group.

Animals and other research organisms

Policy information about studies involving animals; ARRIVE guidelines recommended for reporting animal research, and Sex and Gender in Research

Laboratory animals	This study did not involve laboratory animals.
Wild animals	This study did not involve wild animals.
Reporting on sex	Not applicable as yeast was used as the sole model organism in this study.
Field-collected samples	This study did not involve field-collected samples.
Ethics oversight	No ethical approval or guidance was required as yeast was used in this study.

Note that full information on the approval of the study protocol must also be provided in the manuscript.
